# Acute hemorrhagic conjunctivitis due to enterovirus 70 in India.

**DOI:** 10.3201/eid0502.990212

**Published:** 1999

**Authors:** R S Maitreyi, L Dar, A Muthukumar, M Vajpayee, I Xess, R B Vajpayee, P Seth, S Broor

**Affiliations:** All India Institute of Medical Sciences, New Delhi, India.

## Abstract

An outbreak of acute hemorrhagic conjunctivitis occurred in Delhi, India, during August and September 1996. The etiologic agent was confirmed as enterovirus type 70 by a modified centrifugation-enhanced culture method followed by immunofluorescence and neutralization tests. After nearly a decade, this virus is reemerging as a cause of acute hemorrhagic conjunctivitis in India.


### Acute Hemorrhagic Conjunctivitis Due to Enterovirus 70 in India

**R.S. Maitreyi, L. Dar, A. Muthukumar, M. Vajpayee, I. Xess, R.B. Vajpayee, P. Seth, and S. Broor**
All India Institute of Medical Sciences, New Delhi, India

An outbreak of acute hemorrhagic conjunctivitis occurred in Delhi, India, during August and September 1996. The etiologic agent was confirmed as enterovirus type 70 by a modified centrifugation-enhanced culture method followed by immunofluorescence and neutralization tests. After nearly a decade, this virus is reemerging as a cause of acute hemorrhagic conjunctivitis in India.

Acute conjunctivitis can be caused by viruses including enterovirus 70 (EV-70), coxsackievirus A24, and epidemic adenoviruses. These viruses may also lead to acute hemorrhagic conjunctivitis (AHC), characterized by photophobia, watering, and foreign body sensation, eyelid edema, conjunctival hemorrhages, and superficial punctate keratitis. The disease is self-limiting.

In 1996, an outbreak of AHC occurred in Delhi, north India, during the rainy season (August and September). We conducted a study to identify the etiologic agent by viral culture, immunofluorescence and neutralization tests, and polymerase chain reaction (PCR).

### The Study

We enrolled 13 patients with clinically diagnosed bilateral AHC who attended the outpatient clinic of Rajendra Prasad Centre for Ophthalmic Sciences during the outbreak. At the initial visit, all patients had a complete ophthalmic examination, including slit-lamp biomicroscopy. Conjunctival swabs were taken from the right eye of each patient. These swabs were collected in 2 ml of Hanks balanced salt solution with antibiotics and were transported on wet ice to the virology laboratory for processing.

All samples were vortexed thoroughly and treated with antibiotics (1,000 IU/ml penicillin and 1,000 µg/ml streptomycin). Specimens were clarified by centrifugation at 700 x g for 10 minutes at 4°C. Supernatant fluid was separated and used for inoculation in cell culture. The samples were stored at -70°C until they were processed.

For virus culture, Hep-2–cell monolayers were grown in 24-well plates, containing 12-mm cover slips (Bellco Glass Inc., New Jersey, USA). Each plate was seeded with 1 ml of Hep-2–cell suspension, incubated at 37°C in a 5% $CO_2$ atmosphere, and used for a modification of the centrifugation-enhanced viral culture technique (1).

Cover-slip monolayers were washed twice with phosphate-buffered saline (PBS) before specimen inoculation. Two hundred microliters of each specimen was inoculated in parallel in two 24-well plates containing Hep-2–cell monolayers grown onto 12-mm cover slips. Plates were centrifuged at 700 x g for 60 minutes at room temperature. Inoculum was discarded and washed once with PBS; infected cells were re-fed with 1.0 ml of Eagle's minimum essential medium containing 2% fetal calf serum. The plates were reincubated at 37°C in a 5% $CO_2$ atmosphere. Cover slips from one plate were removed, washed twice with PBS, and fixed with chilled acetone at 48 hours postinoculation for immunofluorescence. The parallel 24-well plate cultures were observed for viral cytopathic effect, which included rounding and refractility of cells and destruction of the monolayer at 2 to 4 days; the resulting effect suggested enteroviral infection. Because a standardized reverse transcriptase-PCR test for enteroviral RNA was available at the All India Institute of Medical

Address for correspondence: Shobha Broor, Department of Microbiology, All India Institute of Medical Sciences, Ansari Nagar, New Delhi-110029, India; fax: 91-11-686 2663; e-mail: broor@medinst.ernet.in.

Sciences, New Delhi, India, we screened the samples with this test.

Ten of the 13 clinical samples and the positive control showed the amplicon band, while the negative control did not (Figure). The PCR results suggested enteroviral infection, helped narrow the search for the etiologic agent, and provided a rapid preliminary diagnosis.

Viral antigen was detected by indirect immunofluorescence staining (on the fixed cover slips stored earlier) using specific antibodies to EV70, coxsackievirus A24, and adenoviruses (Chemicon International Inc., CA, USA).

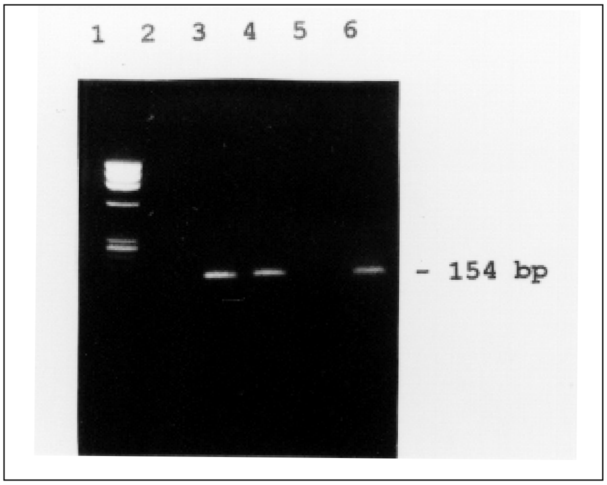

Figure: Reverse transcriptase-polymerase chain reaction (RT-PCR) for enteroviral RNA from acute hemorrhagic conjunctivitis cases. Lane 1: pX174 DNA/Hae III marker; lane 2: negative control; lane 3: positive control (enteroviral RNA); lanes 4-6: clinical samples.

For PCR, viral RNA was extracted by the guanidinium thiocyanate method (2) from 200 µl of clinical specimen. The RNA pellet was resuspended in 10 µl of diethyl pyrocarbonate treated water. A 5-µl volume was used for cDNA synthesis. The primers used for PCR amplification were selected from the highly conserved 5' noncoding region of enterovirus described by Rotbart et al. (3) and, using the same protocol, cDNA for PCR was synthesized by reverse transcription. Briefly, cDNA (10µ Cl) was amplified in a PCR mix volume of 50 µl, containing 100 ng of each primer, 250 µl of each dNTP, 1.5 mM $MgCl_2$, 2.5 units Taq polymerase, and 5 µl of 10X PCR buffer. The tubes were subjected to 30 cycles at 94°C for 1 minute, 58°C for 1 minute, and 72°C for 2 minutes, respectively, followed by a final extension for 7 minutes at 72°C. A 154-bp sized band of the PCR-amplified product was visualized under UV illumination on a 2% agarose gel after ethidium bromide staining.

Infected cover-slip monolayers were stained with 25 µl of monoclonal antibodies, incubated in a moist chamber at 37°C for 45 minutes, and washed twice with PBS for 10 minutes each. Fluorescein-labeled anti-mouse conjugate was added, and the cover slips were reincubated at 37°C for 45 minutes, followed by a PBS washing step, as described above. The cover slips were air-dried, mounted with glycerol buffer, and examined under fluorescence microscopy.

The samples showing a viral cytopathic effect were given a second passage, and if the effect was seen again, the 50% tissue infective dose of the virus isolate was calculated. A virus neutralization test was performed using 100 50% tissue infective doses of the isolate and virus-specific antiserum.

### Findings

All 13 patients enrolled in the study (9 male and 4 female, ages 14 to 37 years) had bilateral ocular involvement and described redness and watering of the eyes, mild photophobia, and severe foreign body sensation. Ocular examination showed severe conjunctival congestion, interspersed subconjunctival hemorrhages, and superficial punctate epithelial keratitis. No neurologic manifestations (as reported in an earlier epidemic) were observed (4).

Cover-slip monolayers infected with a known EV-70 prototype (Kono)–like strain from a previous outbreak (5) and 10 of the 13 clinical specimens showed specific cytoplasmic fluorescence with monoclonal antibodies to EV-70. Infected monolayers tested with monoclonal antibodies to adenovirus and coxsackievirus A24 yielded negative results. Neutralization test results confirmed the identification, as all 10 clinical isolates were neutralized by EV-70–specific antisera. Also, the known EV-70 prototype (Kono)–like strain was neutralized by pooled convalescent-phase sera from the patients in this outbreak. PCR was positive for EV-70 in 11 of the 13 cases, including the 10 culture-confirmed cases.

### Conclusions

An outbreak of AHC was first reported from Ghana in 1969 and was referred to as Apollo conjunctivitis (6). A new enterovirus (EV-70) was identified as the etiologic agent of AHC (7); subsequently it spread to other parts of Africa and Asia including India.

The first serologic evidence of EV-70 infection in India came from Bombay (western India) in 1971-72 (4), and the first isolation was reported from a single case during a small epidemic in southern India in 1975 (8). During an epidemic in north India in 1981 (also during the rainy season), two isolates of EV-70 prototype (Kono)–like strain were reported from Delhi (5), and antigen-positive cases were found by immunofluorescence in the city of Chandigarh (9). The last reported outbreak of EV-70 from this region (July to September 1986) was also confirmed only by demonstration of antigen in cell scrapings by immunofluorescence (10). AHC due to EV-70 appears to have reemerged in north India after nearly a decade. During the intervening period, a coxsackievirus A24 variant was circulating as a cause of AHC in this region of India (11). However, a prime-type EV-70 isolate was obtained from a case-patient during an outbreak in Pune, western India, nearly 1,200 km from Delhi, in 1991 (12).

In this outbreak of AHC, we achieved an unusually high isolation rate of EV-70 (10 of 13 cases) by using a modification of centrifugation-enhanced culture. The results of neutralization tests indicate that the strains circulating in this part of India continue to resemble the prototype strain (also reported during the 1981 epidemic). PCR was useful because of its rapidity and help in narrowing the search for an etiologic agent to enteroviruses. With the availability of PCR based on EV-70 specific primers (13), this highly sensitive centrifugation-enhanced technique is likely to be used increasingly in the future.

Dr. Maitreyi is a senior research fellow, Department of Microbiology, All India Institute of Medical Sciences, New Delhi, India. Her areas of expertise include tissue culture and molecular biology, and her research focuses on respiratory viruses and enteroviruses.

### References

1. Pass RF, Britt WJ, Stagno S. Cytomegalovirus. Diagnostic procedures for viral, rickettsial and chlamydial infections, 7th ed. In: Lennette EH, Lennette DA, Lennette ET, editors. Washington: American Public Health Association; 1995.
2. Chomczynski P, Sacchi N. Single step method of RNA isolation by acid guanidinium thiocyanate phenol chloroform extraction. Ann Clin Biochem 1987;162:156-9.
3. Rotbart HA. Enzymatic amplification of the enteroviruses. J Clin Microbiol 1990;28:438-42.
4. Kono R, Miyamura K, Tajiri E, Shiga S, Sasagawa A, Irani PF, et al. Neurological complications associated with acute haemorrhagic conjunctivitis virus infection and its serological confirmation. J Infect Dis 1974;129:590-3.
5. Manjunath N, Balaya S, Mahajan VM. Isolation of enterovirus 70 during conjunctivitis epidemic in Delhi in 1981. Indian J Med Res 1982;76:653-5.
6. Chatterjee S, Quarcoopome CO, Apenteng A. Unusual type of conjunctivitis in Ghana. Br J Ophthalmol 1970;54:628-30.
7. Kono R, Sasagawa A, Ishii K, Sugiura S, Ochi M, Matsumiya H, et al. Pandemic of new type of conjunctivitis. Lancet 1972;i:1191-4.
8. Christopher S, John J, Charles V, Ray S. Coxsackie virus A24 variant EH 24/70 and enterovirus type 70 in an epidemic of acute haemorrhagic conjunctivitis-a preliminary report. Indian J Med Res 1977;65:593-5.
9. Pal SR, Szucs Gy, Melnick JL, Kaiwar R, Bharadwaj G, Singh R, et al. Immunofluorescence test for the epidemiological monitoring of acute haemorrhagic conjunctivitis cases. Bull World Health Organ 1983;61:485-90.
10. Kishore J, Manjunath N, Bareja U, Verma LK, Broor S, Seth P. Study of an outbreak of epidemic conjunctivitis in Delhi in 1986. Indian J Pathol Microbiol 1989;32:266-9.
11. Broor S, Kishore J, Dogra V, Satapathy G, Seth P. An epidemic of acute haemorrhagic conjunctivitis caused by Coxsackie A24 variant. Indian J Med Res 1992;95:253-5.
12. Bhide VS, Prasad SR, Gogate SS. Isolation of a variant of enterovirus 70 from a patient during an epidemic of acute haemorrhagic conjunctivitis in Pune in 1991. Acta Virol 1994;38:245-6.
13. Uchio E, Yamazaki K, Aoki K, Ohno S. Detection of enterovirus 70 by polymerase chain reaction in acute haemorrhagic conjunctivitis. Am J Ophthalmol 1996;122:273-5.
